# Adolescents' Resilience During COVID-19 Pandemic and Its Mediating Role in the Association Between SEL Skills and Mental Health

**DOI:** 10.3389/fpsyg.2022.801761

**Published:** 2022-02-07

**Authors:** Ilaria Grazzani, Alessia Agliati, Valeria Cavioni, Elisabetta Conte, Sabina Gandellini, Mara Lupica Spagnolo, Veronica Ornaghi, Francesca Micol Rossi, Carmel Cefai, Paul Bartolo, Liberato Camilleri, Mollie Rose Oriordan

**Affiliations:** ^1^“R. Massa” Department of Human Sciences for Education, University of Milano-Bicocca, Milan, Italy; ^2^University of Malta, Msida, Malta

**Keywords:** mental health, social-emotional learning, resilience, adolescents, externalizing behaviors, internalizing behaviors, prosocial behaviors

## Abstract

The main purpose of this paper is to investigate the role of social and emotional learning (SEL) skills and resilience in explaining mental health in male and female adolescents, during the COVID-19 pandemic. Three self-report questionnaires were administered to 778 participants aged between 11 and 16 years (mean age = 12.73 years; SD = 1.73) and recruited from 18 schools in Northern Italy. The SSIS-SEL*b*-S and the CD-RISC 10 assessed SEL and resilience skills respectively, while the Strengths and Difficulties Questionnaire (SDQ) was used to measure mental health in terms of internalizing problems, externalizing problems, and prosocial behavior. We found that SEL and resilience skills were positively and significantly associated with each other, negatively associated with internalizing and externalizing problems, and positively related to prosocial behavior. Three linear regression analyses showed the significant role of resilience, age, and gender in explaining the variance of internalizing problems; the significant role of SEL skills, resilience, age, and gender in explaining the variance of externalizing problems; and the role of SEL skills, age, and gender in explaining prosocial behavior. Importantly, we found that resilience fully mediated the relationship between SEL skills and internalizing problems, partially mediated the relationship between SEL skills and externalizing problems and didn't mediate the relationship between SEL skills and prosocial behavior. The paper concludes with a discussion of the limitations of the study as well as its practical implications.

## Introduction

The aim of this study was to investigate the role of protective factors such as social and emotional learning (SEL) and resilience skills on the mental health of adolescents. This age-group is particularly vulnerable because of their demanding developmental tasks, such as construction of identity, critical and moral thinking, and romantic relationships. The implementation of these tasks has been extremely exacerbated by the COVID-19 pandemic (Singh et al., 2020).

Over the past 20 years, the study of adolescence as a crucial stage of lifespan development has become one of the most important issues in developmental psychology (Hendry and Kloep, 2002; Cavioni et al., 2021). Plenty of studies have shown that identity construction during adolescence is a complex process connected to individual, social, and contextual factors that influence the developmental trajectories, both typical and atypical, from early to late adolescence (Grazzani et al., 2011; Ferrer-Wreder and Kroger, 2019).

Recent research pointed out an increased number of mental health problems among adolescents (Erskine et al., 2015; WHO, 2018, 2020). The incidence of diagnosable mental health problems is approximately around 20%, with half of all mental health issues starting at 14 years of age; moreover, anxiety, depression, eating disorders, addictive disorders, suicidal attempts, and self-harm being the most common mental health issues (Green et al., 2005; Gore et al., 2011; Twenge et al., 2018; Burstein et al., 2019). Recent studies estimated that most of such cases remain undetected and only one-third of these young people receive the necessary psychological support (Merikangas, 2018; Deighton et al., 2019; Twenge, 2020). During the COVID-19 period, adolescents have been severely and negatively affected by the lockdown measures namely social distancing and the closure of schools (Viner et al., 2020). Feeling of stress, anxiety, worries, helplessness, depression, and lack of motivation have been frequently reported among adolescents as a consequence of the pandemic (Fegert et al., 2020).

Recent literature on risk and protective factors has shown that SEL and resilience influence well-being and mental health from the preschool years onward (Cefai et al., 2018; Cahill and Dadvand, 2020; Cavioni et al., 2020a). Nevertheless, few studies have focused on either of these variables during adolescence or on their combined contribution to explain variability in reported mental health during the COVID-19 pandemic. Thus, we look at this recent and emerging literature to build up the rationale of the present study.

### SEL and Mental Health in Adolescence

SEL is defined as the process through which children, young people and adults acquire and apply the knowledge, skills, and attitudes to develop healthy identities, manage emotions and achieve personal and collective goals, feel, and show empathy for others, establish, and maintain supportive relationships, and make responsible and caring decisions (CASEL, 2020; Mahoney et al., 2020). According to the CASEL model, SEL is composed of five core inter-related competences namely self-awareness, self-management, social awareness, relationship skills, and responsible decision-making. Supporting SEL competences in adolescence (Williamson et al., 2015; Hurrelmann and Quenzel, 2018) can be particularly worthwhile as young people are required to develop their personal and social identities (self-awareness), regulate complex emotions and behaviors appropriately (self-management), improve their perspective-taking skills for interpreting social cues accurately (social awareness), effectively negotiate relationships and conflicts with peers and adults (relationship skills) and make ethical decisions about daily challenges contributing to one's own and others well-being (responsible decision making).

Comprehensive, universal, and multi-year SEL initiatives have been largely implemented in the last three decades (Durlak and Wells, 1997; Catalano et al., 2002; Ross and Tolan, 2018). Regarding adolescents, outcomes indicated that SEL has twofold benefits: first, the promotion of mental health; and second, the prevention of psychological problems. With regard to mental health outcomes, the comparison between pre- and post-test scores of experimental and control populations showed improved social-emotional skills, including self-control, decision-making, communication, and problem-solving skills, as well as more positive attitudes about self and others (Payton et al., 2008; Sklad et al., 2012; O'Connor et al., 2018). Furthermore, several studies showed that SEL programs yielded benefits in reducing the likelihood of internalizing—e.g., stress, anxiety, depression, social withdrawal, self-harm, suicidal thoughts, and suicide—and externalizing problems such as conduct problems, impulsivity, violence, crime, high-risk sexual behaviors, and substance use (Greenberg et al., 2003; Browne et al., 2004; Weare and Nind, 2011; Wallender et al., 2020). Notably, follow-up studies indicated these effects persisted over time (Taylor et al., 2017).

Previous studies have shown gender differences in adolescents' mental health, with girls displaying more internalizing behaviors (e.g., related to anxiety, depression, and somatic complaints) and males showing greater externalizing behaviors (e.g., concerning aggressive and delinquent conducts; see, for instance, van der Ende and Verhulst, 2005). In the meta-analysis by Durlak et al. (2011), it was also noted that gender is a factor that may influence the impact of SEL programs on mental health. For instance, in the study by Taylor et al. (2002), boys gained more benefit in self-management and reduced aggressive behaviors compared to females. Although social and emotional skills seem to operate differently on mental health outcomes for girls and boys, research is still needed to further examine the role of gender in explaining how SEL competences impact on mental health.

Recent evidence also suggests that social and emotional competences tend to decline from childhood to adolescence (Ciucci et al., 2016; West et al., 2018; Green et al., 2021). However, studies focusing on how the core five SEL skills develop throughout adolescence and how they impact on mental health are still limited (West et al., 2020; Farina et al., 2021) with research tending to mainly explore social and emotional skills and mental health during early childhood or primary school periods (e.g., Ahlen et al., 2015). Therefore, there is a clear need for research testing the empirical SEL model in adolescent populations, its relationship with mental health outcomes, and how this relationship may change across years during adolescence (Ross and Tolan, 2018; West et al., 2020).

### Resilience and Mental Health in Adolescence

Resilience is defined as the dynamic capacity, processes, or outcomes of successful adaptation in the context of significant threats to function or development (Rutter, 1999; Masten, 2011). Research has identified resilience as a complex construct resulting from a dynamic relationship between risk and protection factors in which individuals may use personal and contextual resources to overcome adversities (Luthar et al., 2000; Ungar, 2018; Höltge et al., 2021). Thus, resilience serves to protect and promote individuals' psychological well-being mitigating the negative effects of stressful events, accelerating the recovery, and reducing the risk of developing mental health problems (Davydov et al., 2010). Resilience may vary according to age due to changes occurring during the lifespan (Masten, 2001; Masten and Barnes, 2018). In adolescence, key assets for resilience include coping skills, stress management, and self-efficacy (Connor and Davidson, 2003), which can help deal with adversities and setbacks, rejection, family conflict, loss, bullying and peer conflicts, life changes and life transitions while protecting young people from the negative consequences associated with exposure to risk situations (Fergus and Zimmerman, 2005; Cefai et al., 2015). Recent work underlined the connection between resilience and mental health, suggesting that more resilient adolescents are less prone to mental health problems, including depression and anxiety problems (Hjemdal et al., 2011; Fischer et al., 2019; Ollmann et al., 2021).

Studies exploring the relationship between resilience and mental health in the light of gender differences suggest greater vulnerability in girls, namely depressive and anxiety symptoms (e.g., Nolen-Hoeksema et al., 1999; Wichstrøm, 1999). These findings have been discussed by Hjemdal et al. (2006, 2011), arguing that adolescent girls seem to be more exposed to stress from their social environment, showing a lower sense of mastery in their lives. Therefore, the gender differences in mental health outcomes may be influenced by the different gendered patterns in facing the challenges of the social environment.

Though resilience is a widely used construct, the relationship between resilience and mental health among adolescents has been scarcely explored in healthy adolescents. In the context of the current COVID-19 pandemic, this association deserves special attention. In fact, the experienced prolonged stressful situation may have shaped the adolescents' capacity to effectively cope with stress and respond to challenges and, in turn, this may have positively or negatively affected their mental health (Loades et al., 2020; Masten and Motti-Stefanidi, 2020).

### SEL Skills and Resilience in Adolescence

Previous research has largely documented that SEL provides opportunities to develop resilience skills to face stressors and difficulties across the life span (Domitrovich et al., 2017). As such, several studies have examined the relation between the five key SEL competences and resilience (Cavioni et al., 2018; Cahill and Dadvand, 2020).

For instance, having a growth mindset is an essential self-awareness ability. The extensive body of research by Dweck (e.g., Dweck, 2006) on the implicit theories of intelligence underlined that students who hold growth mindsets interpret challenges, setbacks and failures as opportunities to improve their learning and skills, believing that they can learn and succeed if they persist. Consequently, adolescents who exhibit higher levels of self-awareness are more likely to successfully face significant life challenges persevering despite difficulties. Furthermore, they tend to show better self-efficacy and hope, which in turn may enhance the ability to effectively recover from traumatic situations (Meng et al., 2018).

The link between self-management and resilience is also well-documented. In adolescence, the ability to regulate emotions and behaviors to pursue specific goals has been found to contribute to resilience (Dishion and Connell, 2006). The positive adaptation to, and successful coping with difficulties have been related to the ability to regulate emotions and behaviors, to reflect critically and to apply effective strategies to moderate or remove the negative effects of stress (Compas et al., 2001). Therefore, higher levels of self-management, including monitoring, and adapting emotional responses, behaviors, emotions, and cognitive strategies, contribute to improving the ability to achieve the desired goals in adverse situations.

Social awareness encompasses various abilities including showing empathy and prosocial behaviors toward others and being able to value and understand others' perspective, a skill present since childhood (Grazzani et al., 2017; Conte et al., 2018; Ornaghi et al., 2020). According to Brooks and Goldstein (2003), empathy is a key component of resilience because by placing yourself in others' situations and understanding others' emotions and thoughts, one can learn and apply resilience without being personally involved into stressful situations. Various studies have explored the relation between social awareness and resilience in adults (e.g., Smith and Hollinger-Smith, 2015), however, this relationship, has been scarcely explored in adolescence. Thus, research is needed to investigate the contribution of social awareness to resilience during adolescence.

Relationship skills include the ability to establish and maintain positive relations, to collaborate and constructively solve conflicts and seek or offer help when needed. Having trusted relationships with others (e.g., peers, family members, teachers) and perceived social support act as protective mechanisms in successfully facing difficult situations (Rutter, 1999). Furthermore, in adolescent girls, closed relationships with peers better facilitate resilience when compared with boys (Graber et al., 2016).

Finally, responsible decision-making is also positively related to resilience. For instance, Smokowski et al. (2000) noted that resilient adolescents develop and apply effective strategies and coping skills to adapt to stressors in decision-making tasks. Furthermore, the capacity of adolescents to reflect on the ethical consequences of their actions on personal, social, and collective well-being is associated with reduced rates of at-risk behaviors (Cahill and Dadvand, 2020).

### The Present Study

In view of this empirical background, the present study attempts to fill a gap in the literature, examining how SEL and resilience skills contribute to mental health in adolescence. On one hand, the relationship between SEL skills and mental health has been scarcely investigated in adolescents (e.g., West et al., 2020; Farina et al., 2021). On the other hand, resilience is usually explored in disadvantaged and atypical populations which cope with difficult adaption (e.g., Masten, 2011), whereas the COVID-19 pandemic has been certainly a special period to examine how resilience skills are activated and impact on mental health in a typical population of adolescents. Thus, we set the three following objectives.

The first objective was to test the existence of associations between SEL skills, resilience, and mental health. We hypothesized that SEL and resilience skills had negative associations with internalizing and externalizing problems, and positive associations with prosocial behavior. In addition, in accordance with previous studies we expected to find differences in SEL and resilience skills and mental health as a function of age and gender.

The second objective was to investigate the role of SEL and resilience skills on adolescents' mental health, controlling for age and gender. Specifically, we hypothesized that SEL and resilience skills impacted mental health, and gender contributed to it, with females displaying more internalizing problems and males more externalizing problems.

The third objective was to examine whether resilience mediated the relationship between SEL skills and mental health amongst adolescents, exploring the hypothesis that resilience was a mediator in the association between SEL skills and internalizing problems, externalizing problems, and prosocial behavior.

## Method

### Participants

The sample comprised 778 adolescents (413 females) aged from 11 to 16 years (*M* = 12.73 years; *SD* = 1.73). They were grouped into two age-groups: 59.4% (aged between 11 and 12 years) attended lower secondary school, and 40.6% (aged between 13 and 16 years) attended high secondary school. This sample guarantees a maximum margin of error of 3.5% assuming a 95% confidence level. The participants were recruited from 18 schools located in medium SES, urban areas in northern Italy, and were stratified by gender and school level. Thus, cluster sampling was used to select the schools ensuring good geographical representation, while stratified sampling was used to select the students from several classrooms within the selected schools. The study was conducted in conformity with the recommendations of the University of Milano—Bicocca Ethics Committee. Parental informed consent was obtained for all participants in line with the Declaration of Helsinki. Participants were free to withdraw from the study at any time, and no monetary or other financial rewards were provided.

### Measures

The participants completed the online Italian self-report versions of three questionnaires aimed at assessing adolescents' social-emotional learning, resilience, and mental health, respectively. The instruments were selected on the basis that they are validated measures and suited to the age of the participants.

Social Skills Improvement System, Social Emotional Learning Edition Brief Scales – Student Form (SSIS-SEL*b*-S) (Elliott et al., 2020). This is a self-report measure of social and emotional learning of students in grades 3-12. It is built on the theoretical model of CASEL, consisting of five SEL domains: self-awareness, self-management, social awareness, relationship skills, and responsible decision making. Overall, it includes 20 items rated on a 4-point scale ranging from 0 (not true) to 3 (very true). The composite score can range from 0 to 60. Each of the five subscales consists of 4 items, with a possible range of scores between 0 and 12. The original instrument has strong reliability, with Cronbach's alphas of 0.91 for the composite score and ranging from 0.67 to 0.72 across the five subscales (Anthony et al., 2020). In the current study, Cronbach's alpha was 0.83 for the composite scale and ranged from 0.50 to 0.75 for the five subscales. Only the composite score was used in this work.

Connor Davidson Resilience Scale (CD-RISC 10; Campbell-Sills and Stein, 2007). This is a self-report measure of resilience in adolescents and adults. The short version of the scale consists of 10 items on a 5-point Likert scale, from 0 (not true at all) to 4 (true nearly all the time). The total score can range from 0 to 40. The Italian version of the tool has demonstrated good internal consistency (Cronbach's alpha = 0.89, Di Fabio and Saklofske, 2014). In the current study, the Cronbach's alpha was 0.84.

Strengths and Difficulties Questionnaire (SDQ; Goodman, 1997). The SDQ is a brief questionnaire for assessing 3–16-year-olds' mental health. For 11-year-olds and older, the self-report version can be used (Goodman et al., 1998). The SDQ consists of 25 items rated on a 3-point Likert scale, where 0 = “not true,” 1 = “somewhat true,” and 2 = “certainly true.” As recommended by Goodman et al. (2010), three scores can be calculated for general population samples: Internalizing Problems (10 items, which include emotional symptoms and peer relationship problems; range of scores: 0-20), Externalizing Problems (10 items, which include conduct problems and hyperactivity/inattention difficulties; range of scores: 0-20), and Prosocial Behavior (5 items; range of scores: 0-10). In the original instrument, Cronbach's alphas were 0.66, 0.76, and 0.66 for Internalizing, Externalizing, and Prosocial scales, respectively (Goodman et al., 2010). In the current study, the Cronbach's alphas were 0.75 for the Internalizing scale, 0.76 for the Externalizing scale, and 0.70 for the Prosocial scale.

### Overview of Analysis

Prior to conducting the main analyses, the data were checked for univariate normality (i.e., distribution, kurtosis, and skewness). The internalizing and externalizing problems and prosocial behavior score distributions were skewed and did not satisfy the normality assumption. To address this limitation, bootstrap standard errors and confidence intervals were provided to account for intrinsic asymmetry and non-Gaussian trends in the regression model. Unlike parametric approaches, bootstrapping resamples a single dataset to create many simulated samples without making any assumptions for the population distribution. This process enables researchers to calculate standard errors, construct confidence intervals and perform hypothesis testing for various types of sample statistics. Next, the main descriptive statistics and zero-order correlations were computed.

Regarding the second and third objectives of the study, three general linear regression models were fitted to relate each of the three outcome variables (internalizing and externalizing problems and prosocial behavior) to four predictors (gender, age, SEL and resilience skills). Regression model measures the impact of each predictor on the dependent variable, while holding the other predictors constant. It relies on several assumptions, including linearity, homoscedasticity, independence and normality that are often violated. For each model, a 0.05 level of significance will be adopted to determine statistical significance. Baron and Kenny (1986) describe a four-step procedure to test for mediation by fitting three regression models. In this paper, the first model relates the outcome variable to SEL skills; the second model relates resilience (mediator) to SEL skills; and the third model relates the outcome variable to both resilience and SEL skills collectively. The Sobel test is then used to determine whether the indirect effect between the dependent variable and SEL skills via resilience is significant, using a 0.05 level of significance. The software used for all analyses was IBM SPSS Statistic 27.

## Results

Descriptive statistics and zero order correlations for the variables under study are reported in Table 1. The correlational analysis revealed that internalizing and externalizing problems are positively and significantly related indicating that adolescents who have peer and emotional problems also tend to have conduct and hyperactivity problems. Moreover, prosocial behavior, SEL skills and resilience are positively and significantly related, indicating that adolescents who are resilient and have good social and emotional competencies tend to exhibit more prosocial behavior. Conversely, as shown in Table 1, internalizing and externalizing problems are negatively and significantly associated to prosocial behavior, SEL skills, and resilience, which implies that adolescents with personal and behavioral difficulties tend to be less prosocial, less resilient and have poorer social and emotional competencies. In addition, internalizing and externalizing problems significantly increased with age whereas SEL skills, resilience, and prosocial behavior were negatively and significantly associated with age. Gender was negatively correlated with resilience and positively correlated with SEL skills, prosocial behavior and internalizing problems, indicating that male participants as compared to females obtained higher scores at resilience and lower scores at SEL skills, prosocial behavior and internalizing problems.

**Table 1:** Means, standard deviations, and zero-order correlations among the study variables.

	***M***	***SD***	**1**	**2**	**3**	**4**	**5**	**6**	
1. SEL	39.19	7.74	-						
2. Resilience	21.97	7.95	0.466^**^	-					
3. Internalizing	5.51	3.79	−0.0216^**^	−0.0494^**^	-				
4. Externalizing	5.96	3.63	−0.466^**^	−0.382^**^	0.519^**^	-			
5. Pros. Behavior	7.60	1.81	0.603^**^	0.319^**^	−0.105^*^	−0.261^**^	-		
6. Age	12.73	1.73	−0.205^**^	−0.179^**^	0.291^**^	0.225^**^	−0.177^**^	-	

* *p < 0.05*,

** *p < 0.001*.

A first regression analysis was carried out to investigate whether adolescents' SEL skills, resilience, age, and gender explained the variance in mental health. The first model relates the internalizing problem score to four predictors (SEL skills, Resilience, Age and Gender). Table 2 displays the parameter estimates and corresponding 95% confidence intervals, standard errors, and biases. This four-predictor model explains 30.8% of the total variation in the internalizing problem score (*R*^2^ = 0.308), where three predictors are significant (Resilience, Age and Gender). The internalizing problem score is expected to increase by 0.416 for every 1-year increase in age and decrease by 0.202 for every 1 unit increase in the resilience score. Moreover, males are expected to score 1.175 less than females, given that other effects are kept constant. Although SEL skills are not significant in this four-predictor model, they become significant on removing resilience from the model fit, suggesting resilience as a full mediator variable.

**Table 2:** Regression outcomes for the target variable Internalizing problems.

					**95% Conf. Interval**	
	**B**	**SE**	***t***	***p***	**Lower bound**	**Upper bound**
Intercept	5.1392	1.2111	4.244	<0.001	2.762	7.517
SEL	−0.0002	0.0177	−0.013	0.990	−0.035	0.035
Resilience	−0.2023	0.0173	−11.67	<0.001	−0.236	−0.168
Age	0.4160	0.0695	5.983	<0.001	0.280	0.553
Gender	−1.1751	0.2486	−4.727	<0.001	−1.663	−0.687

*R^2^ = 0.308, Gender is a categorical predictor, where male = 1, female = 0*.

The second regression relates the externalizing problem score to the four predictors (SEL skills, Resilience, Age and Gender). Table 3 displays the parameter estimates and corresponding 95% confidence intervals, standard errors, and biases. This four-predictor model explains 27.7% of the total variation in the externalizing problem score (*R*^2^ = 0.227), where all four predictors are significant. The externalizing problem score is expected to increase by 0.208 for every 1-year increase in age, decrease by 0.181 for every 1-unit increase in the SEL skills score, and decrease by 0.074 for every 1 unit increase in the resilience score. Moreover, males are expected to score 0.622 less than females, given that other effects are kept constant. The removal of resilience from the model fit makes SEL skills a stronger predictor, suggesting resilience as a partial mediator variable.

**Table 3:** Regression outcomes for the target variable Externalizing problems.

					**95% Conf. Interval**	
	**B**	**SE**	***t***	***p***	**Lower bound**	**Upper bound**
Intercept	12.2529	1.1954	10.25	<0.001	9.906	14.600
SEL	−0.1810	0.0175	−10.36	<0.001	−0.215	−0.147
Resilience	−0.0741	0.0171	−4.330	<0.001	−0.108	−0.041
Age	0.2077	0.0686	3.026	0.003	0.073	0.342
Gender	−0.6216	0.2454	−2.533	0.012	−1.103	−0.140

*R^2^ = 0.227, Gender is a categorical predictor, where male = 1, female = 0*.

The third regression relates the prosocial behavior score to the four predictors. Table 4 displays the parameter estimates and corresponding 95% confidence intervals, standard errors and biases. This four-predictor model explains 37.4% of the total variation in the prosocial behavior score (*R*^2^ = 0.374), where three predictors are significant (SEL skills, Age and Gender). The prosocial behavior score is expected to decrease by 0.056 for every 1-year increase in age and increase by 0.127 for every 1 unit increase in the SEL skills score. Moreover, males are expected to score 0.252 less than females in prosocial behavior, given that other effects are kept constant.

**Table 4:** Regression outcomes for the target variable Prosocial behavior.

					**95% Confidence Interval**	
	**B**	**SE**	**t**	**p**	**Lower bound**	**Upper bound**
Intercept	3.1365	0.5547	5.655	<0.001	2.048	4.225
SEL	0.1274	0.0081	15.72	<0.001	0.111	0.143
Resilience	0.0133	0.0079	1.678	0.094	−0.002	0.029
Age	−0.2083	0.0671	−3.104	0.002	−0.339	−0.071
Gender	−0.2516	0.1138	−2.210	0.027	−0.479	−0.034

*R^2^ = 0.374, Gender is a categorical predictor, where male = 1, female = 0*.

**Figure 1:**
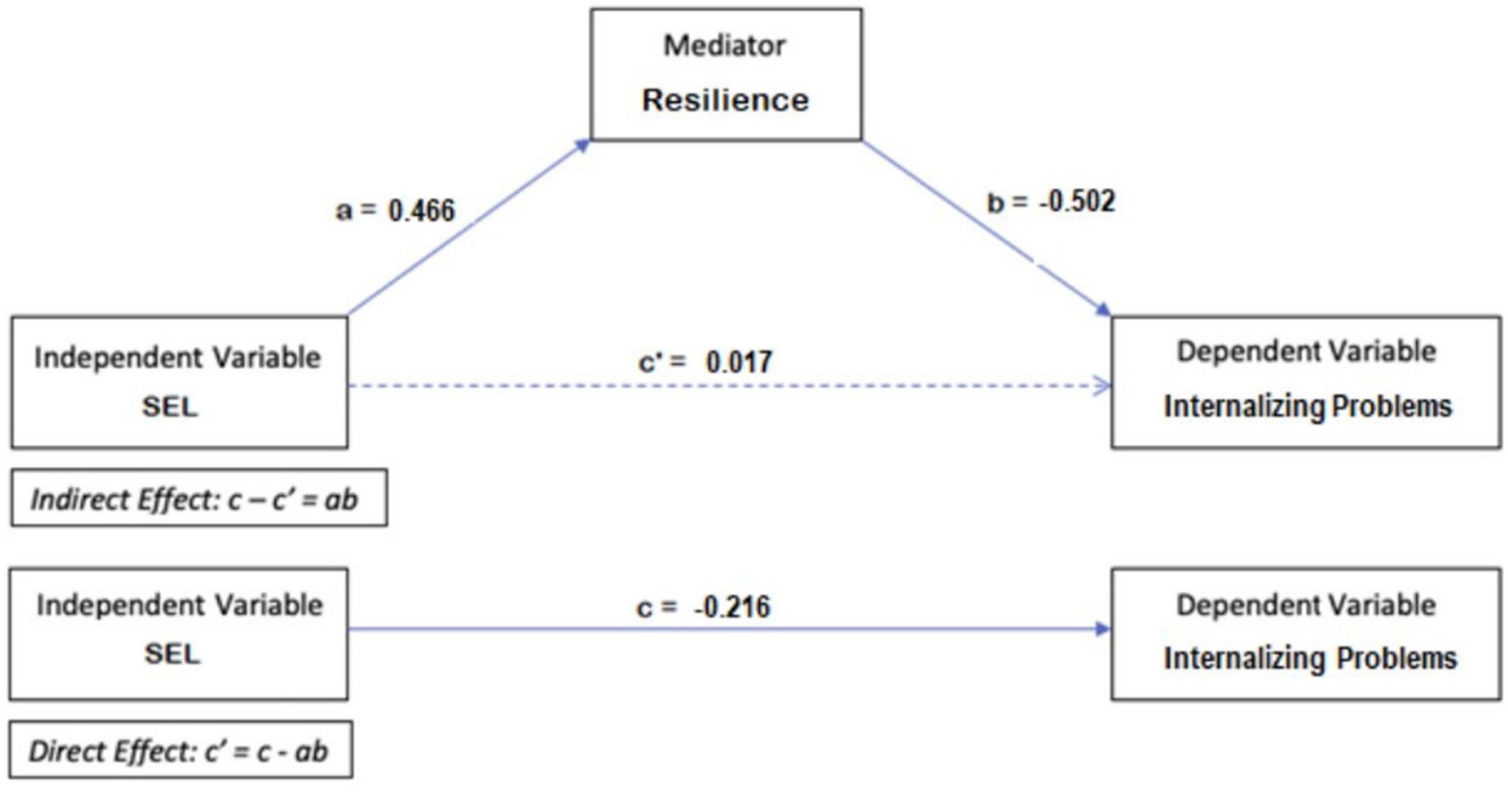
Mediation model for internalizing problems.

A mediation model was then performed to further investigate the association between SEL skills and mental health via the inclusion of resilience as a mediator variable. More precisely, to investigate whether resilience mediates the relationship between SEL skills and internalizing problems, three linear regression models were fitted. A summary of the results of these models is displayed in Figure 1. The first model which relates internalizing problems (dependent variable) to SEL skills (independent variable) yields the total effect size (c = −0.216). The second model which relates resilience (mediator) to SEL skills (independent variable) yields the effect size (a = 0.466; B = 0.474, S.E. = 0.033, t = 14.402, *p* <0.001). The third model which relates internalizing problems to both resilience and SEL skills yields the sizes of the effects (b = −0.502 and c**'** = 0.017; respectively: B = −0.239, S.E. = 0.017, t = −13.945, *p* < 0.001; B = 0.008, S.E. = 0.017, t = 0.467, *p* = 0.641). The Sobel test shows that the indirect effect between SEL skills and internalizing problems via resilience (ab = −0.234) is significant (z = −10.047, S.E. = 0.0113, *p* < 0.001). However, the direct effect (c**'** = 0.017) is not significant. This implies full mediation, which indicates that better SEL skills enhance resilience, which in turn reduces internalizing problems (B = −0.105, S.E. = 0.017, t = −6.062, *p* < 0.001).

**Figure 2:**
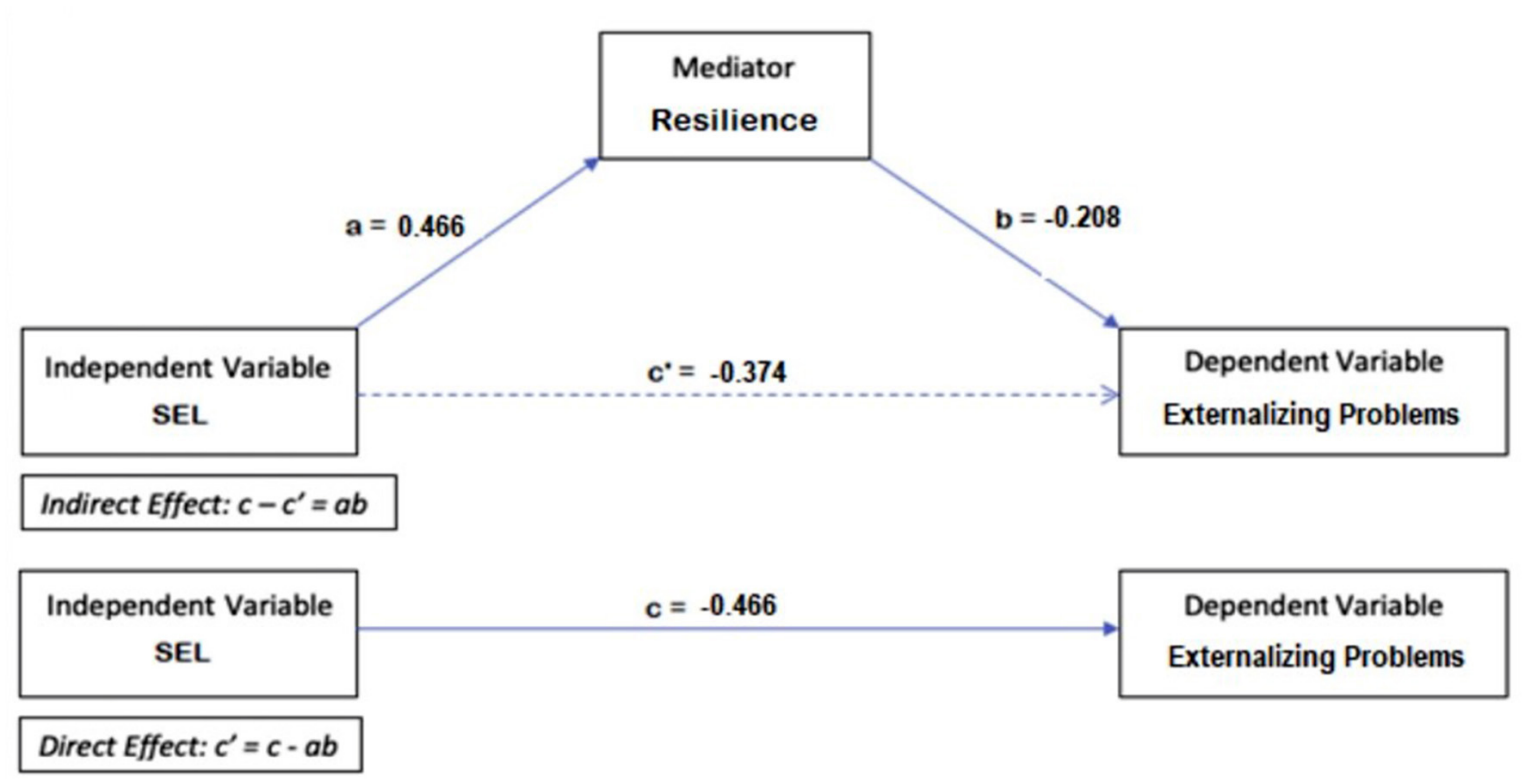
Mediation model for externalizing problems.

A similar procedure was used to investigate whether resilience mediates the relationship between SEL skills and externalizing problems, as shown in Figure 2 (B = −0.217, S.E. = 0.015, t = −14.461, *p* < 0.001). The Sobel test shows that the indirect effect between SEL skills and externalizing problems via resilience (ab = −0.097) and the direct effect (c**'** = −0.374) are both significant (z = −5.438, S.E. = 0.0082, *p* < 0.001). This implies partial mediation, which indicates that both resilience and higher levels of SEL skills are essential to reduce externalizing problems (respectively: B = −0.172, S.E. = 0.016, t = −10.476, *p* < 0.001; B = −0.094, S.E. = 0.016, *t* = −5.924, *p* < 0.001).

**Figure 3:**
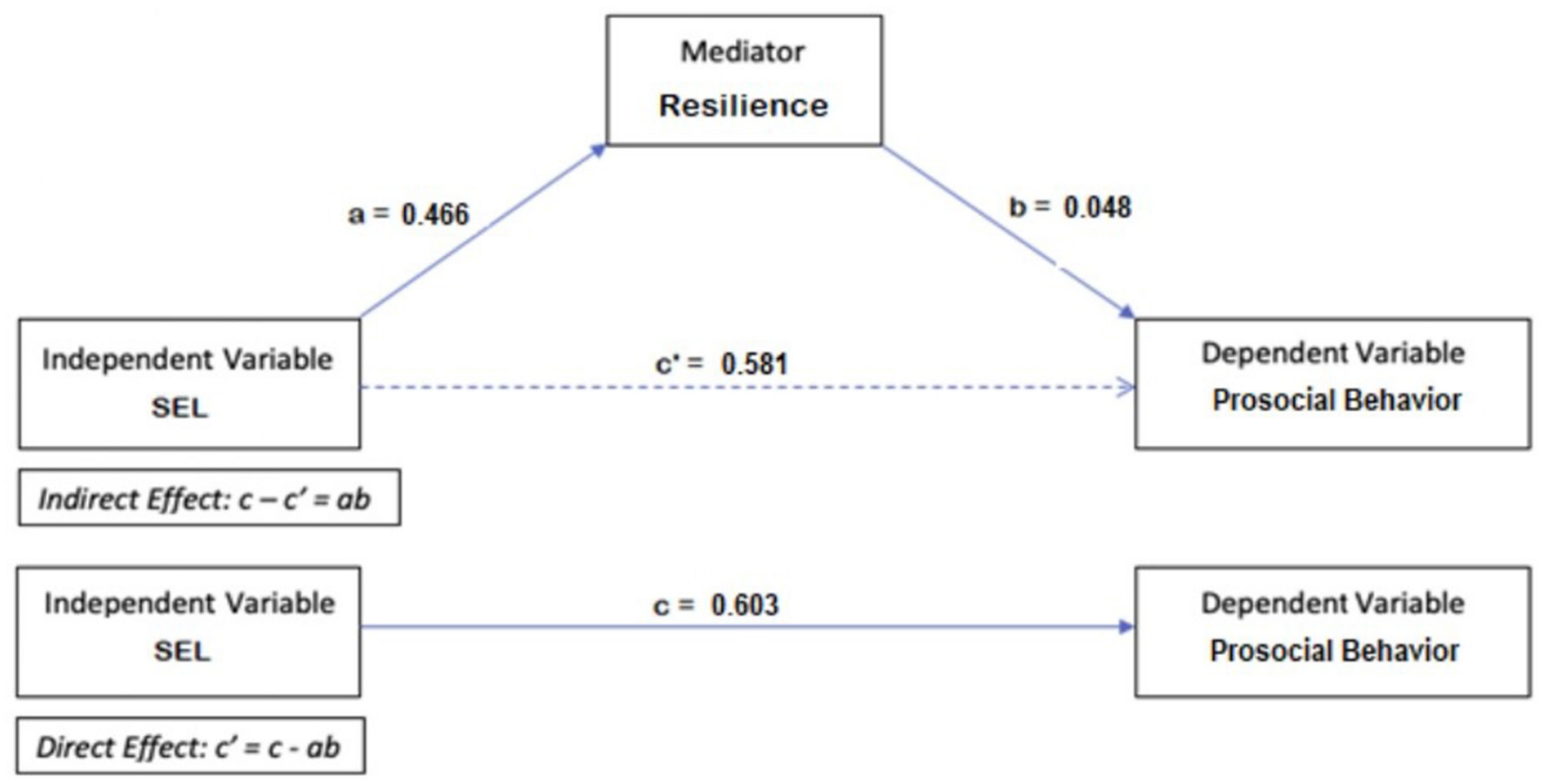
Mediation model for prosocial behavior.

A similar procedure was utilized to investigate if resilience mediates the relationship between SEL skills and prosocial behavior, as shown in Figure 3 (B = 0.140, S.E. = 0.007, t = 20.730, *p* < 0.001). The Sobel test shows that the indirect effect between SEL skills and prosocial behavior via resilience (ab = 0.022) is not significant (B = 0.011, S.E. = 0.007, *t* = 1.456, *p* = 0.146). However, the direct effect (c**'** = 0.581) is significant (z = 1.562, S.E. = 0.0033, *p* = 0.118). This implies no mediation, which indicates that prosocial behavior is improved with better SEL skills, but resilience has negligible impact on this positive relationship.

## Discussion

The aim of the current study was to investigate the relationship between resilience, SEL skills, and mental health, and how this varies by age and gender in an adolescent sample. This topic was investigated during a challenging period, that is the COVID-19 pandemic, in which people were in a position to use and exploit their resilience skills. To the best of our knowledge, this is the first study to have examined such variables in adolescence, with a specific focus on resilience as a mediating factor between SEL skills and mental health. We obtained three main findings. Firstly, SEL skills, resilience and mental health were significantly associated; secondly SEL skills, resilience, age, and gender were found to significantly contribute to explain the variance in mental health; and thirdly, resilience fully mediated the relationship between SEL skills and internalizing problems and partially mediated the relationship between SEL skills and externalizing problems. We discuss these main findings in more detail below.

### Associations Between SEL Skills, Resilience, and Mental Health

The first objective of this study was to examine the associations between SEL skills, resilience, and mental health. As expected, SEL skills and resilience were positively associated, consistent with previous research showing that SEL competences represent a protective factor which helps individuals to be more resilient and adequately face stressors and challenges across the life span (e.g., Durlak et al., 2011; Domitrovich et al., 2017).

Furthermore, both SEL and resilience skills were associated to mental health. Specifically, we found that SEL and resilience skills were negatively related to internalizing and externalizing problems and positively related to prosocial behavior. Evidence-based programs have widely demonstrated that the development of social and emotional competences have 22-fold benefits, namely the prevention of psychological problems and the promotion of mental health (Greenberg et al., 2003; Payton et al., 2008; Sklad et al., 2012; Taylor et al., 2017; O'Connor et al., 2018). Although the association between resilience and mental health has been scarcely explored in healthy adolescents (e.g., Fischer et al., 2019), our findings are consistent with the literature and suggest that more resilient adolescents display more prosocial attitudes and less mental health problems.

### The Role of SEL Skills, Resilience, Age, and Gender on Mental Health

The second aim of this study was to investigate the role of SEL skills, resilience, age, and gender on adolescents' mental health. Results of regression analyses show that resilience, age, and gender explained variance in internalizing problems. This is in line with results of previous studies attesting that higher resilience predicts lower scores of depression, anxiety, and other internalizing problems (Hjemdal et al., 2011; Ollmann et al., 2021). On the other hand, SEL skills did not play a significant role in explaining these problems, suggesting that it is not sufficient to prevent internalizing problems. These findings are consistent with van der Ende and Verhulst's (2005) report of an interaction between age and gender and internalizing problems. Indeed, adolescent girls, compared to boys, self-reported more anxiety and depressive symptoms, and the difference increased with increasing age.

With regard to externalizing problems, we found that SEL and resilience skills are associated with decreased aggressive behavior, violence, and risk behaviors, which is consistent with previous studies (Catalano et al., 2002; Greenberg et al., 2003; Weare and Nind, 2011). Again, with regards to internalizing problems, both age and gender played a significant role. van der Ende and Verhulst (2005) reported that adolescents' externalizing problems increase from 11 to 15 years, reaching a peak at this point, and then they decrease. Since adolescents who participated in our study were aged between 11 and 16 years, it is possible that they self-reported their behaviors during this critical phase when externalizing problems are more likely to be prevalent. Surprisingly, our findings suggest that females were more prone to display externalizing problems than males, which is contrary to our expectations. Externalizing problems are usually more prevalent in boys than girls (van der Ende and Verhulst, 2005). It is likely that adolescent girls were more sensitive to their own and others' negative emotions (e.g., fear, anger, frustration) generated by the COVID-19 pandemic. This may have resulted in higher levels of conduct problems, hyperactivity, and inattention as they struggled to cope with the stress experienced in their life contexts (Bianco et al., 2021).

Finally, SEL skills contributed to prosocial behavior, which is in line with previous studies (Payton et al., 2008; Sklad et al., 2012; O'Connor et al., 2018). The more the adolescent can recognize and manage one's own emotions and behaviors, appreciate others' perspectives, make responsible decisions, and effectively negotiate relationships, the more they will be able to display comforting, sharing, helping, and other positive behavior toward other people. This is particularly true for females, who were inclined to display more prosocial behaviors than males. Girls are generally expected to be more caring and sensitive than boys and this representation drives socialization practices from early stages of life (Eisenberg and Fabes, 1998). During adolescence, physical, cognitive, and social-relational changes lead males and females to progressively adhere to gender role expectations, resulting in gender differences in prosocial behavior (Van der Graaff et al., 2018). Changes in adolescence occur because of hormonal and neural modifications that can also affect adolescents' abilities to regulate their own emotions and to pay attention to goals, needs, and emotions of other people (Crone and Dahl, 2012; Van der Graaff et al., 2018). This could explain why adolescents' prosocial behaviors decreased over time in our study.

### The Mediating Role of Resilience

The third objective of this study was to examine resilience as a mediator between SEL skills and mental health. The study of resilience has been focused on understanding the processes of positive recovery, adaptation, or transformation in contexts of adversity (Ungar, 2018). The data in this study has been collected during the COVID-19 pandemic, which represented an unprecedented challenging time for adolescents' lives. In general, increased prevalence of internalizing problems among adolescents has been largely reported due to the measures taken to reduce the spread of the pandemic, namely lockdown, social distancing, and quarantine measures as well as school closure (Cusinato et al., 2020). Among adolescents, greater levels of stress, depressive and anxiety disorders, both during the initial stage of the pandemic and over time, have been reported (Singh et al., 2020; Wang et al., 2020a,b).

The mediation analysis in our study supported the key role of resilience in mental health. Despite previous research that documented the direct link between SEL competences and mental health (e.g., Durlak et al., 2011; Cavioni et al., 2020b), a potential interesting interpretation of our results is that SEL skills, compared to resilience, may be a weaker protective factor of mental health, as it acted indirectly on internalizing problems during challenging times. It contributed to resilience which, on the other hand, acted directly on adolescents' ability to cope with internalized problems in the challenging pandemic period. Resilience fully mediated the relation between SEL skills and internalizing difficulties, helping to decrease adolescents' depressive symptoms, anxiety, and stress. On the other hand, SEL and resilience skills are both essential in reducing externalizing behavior, while resilience, in contrast to SEL competences, does not impact prosocial behavior. Our study suggests that, in the context of the challenging pandemic context, SEL and resilience skills both impact mental health but through different mechanisms, namely both SEL and resilience skills impact externalizing behavior directly, but whilst SEL competences impact internalizing problems through resilience, resilience has little direct impact on promoting prosocial behavior. Whilst resilience appears to be particularly impactful in reducing mental health difficulties, especially internalizing ones, SEL skills becomes more salient in promoting mental health such as prosocial behavior. These relationships warrant further investigation to establish more clearly how SEL competences and resilience are interrelated to mental health, particularly in times of challenge and stress.

### Limitations, Strengths, and Implications

Three limitations of this study should be noted. Firstly, data were collected with a large sample of Italian adolescents. Consequently, findings need to be generalized with caution to the adolescents' population in other contexts and cultures. Secondly, given the cross-sectional design of this study, caution must be taken when making causal inference from our findings. Thirdly, data were collected via self-report questionnaires only. Therefore, answers provided by the participants might be subjected to social desirability bias. To circumvent these limitations, future research needs to collect data from adolescents coming from different contexts and cultures, include follow up data collection and use a multi-informant approach.

Despite these limitations, the key strengths of the present study are represented by the large sample and the timing of data collection during the COVID-19 pandemic. In fact, this study has the added value of investigating the contribution of resilience to mental health in a sample of healthy adolescents during a challenging time. Recent studies have called for the urgent need to address the increasing concern of mental health in adolescents, also in the light of the growing depressive and anxiety symptoms due to the COVID-19 pandemic (e.g., Idele and Banati, 2021). Our findings suggest that resilience may be an important protective factor against the incidence of such internalized disorders especially in the face of adversity and challenging situations. Therefore, understanding the role of resilience in the present situation of the pandemic becomes crucial to plan adequate interventions and prevent any further increase in psychological distress and negative consequences for the mental health and well-being of adolescents.

## Conclusion

A growing number of studies have been conducted to explore the psychological impact of the COVID-19 on adolescents as a consequence of the prolonged experience of forced social isolation (Tang et al., 2020; De France et al., 2021). Given the increased level of severe mental health problems, it's imperative to further deepen the understanding of the mechanisms of counterbalancing the negative effects of the pandemic to face the psychological vulnerability and distress among adolescents. Our findings suggest a tendency to experience higher internalizing and externalizing problems that progresses with age. Consequently, late adolescents appear to be at heightened risk for developing mental health difficulties during the pandemic. Therefore, it is crucial to address the promotion of resilience specifically among this age group. As Cefai and colleagues noted (Cefai and Cavioni, 2015; Cefai, 2021), resilience should be addressed by adopting a systemic perspective that considers the relationships with family members, social groups, and communities that the adolescents live in, a key protective factor against the development of mental health difficulties. Therefore, an essential component to promote the mental health of adolescents during the pandemic should be maintaining and nurturing supportive social relationships (Zhou, 2020).

The outcomes of the present study contributed to identify resilience as a strong protective factor particularly in reducing internalizing problems. Moreover, a fundamental remark has to be made concerning gender, since we found that adolescent females were more vulnerable to the negative psychological effects of the pandemic, experiencing greater levels of internalizing and externalizing problems than males. Therefore, this study represents a significant contribution not only to explore the relationships among social and emotional learning, resilience and mental health in adolescence but also to better understand the specific impact of the pandemic, suggesting that there may be different manifestations of psychological distress across genders during this highly stressful event. To conclude, the importance of developing resilience and social and emotional competences underpins increasing efforts to start promoting these abilities in the early years of life, as documented by recent innovative lines of research with toddlers (Grazzani et al., 2016a,b; Ornaghi et al., 2019; Brazzelli et al., 2021).

## Data Availability Statement

The raw data supporting the conclusions of this article will be made available by the authors, without undue reservation.

## Ethics Statement

The studies involving human participants were reviewed and approved by the Ethical Committee of the University of Milano-Bicocca. Written informed consent to participate in this study was provided by the participants' legal guardian/next of kin.

## Author Contributions

IG made a key contribution to designing the research, interpreting the data, and drafting and revising the manuscript. AA contributed to the conception and design of the research and to data collection. VC and EC has made substantial contributions to the conception and design of the research study, to the collection of the data, and the drafting and revision of the manuscript. SG contributed to the conception and design of the research and in data collection. ML has been involved in the conception of the study and contributed to collecting the data. VO made a key contribution to designing the research, analyzing, interpreting the data, drafting, and revising the manuscript. FR has been involved in the development of the study and revising the manuscript critically. CC has made substantial contributions to the conception and design of the research, interpreting the data, and revising of the manuscript. PB has been involved in the conception and design of the research, interpreting the data, and revising of the manuscript. LC contributed to the conception and design of the research, analyzing and interpreting the data, and drafting the manuscript. MO has been involved in the conception and design of the research and analyzing the data. All authors read and approved the final manuscript.

## Funding

This study was co-funded by the Erasmus+KA3 programme of the European Union PROMEHS Promoting Mental Health at Schools (Reference number: 606689-EPP_2018-2-IT-PI-POLICY).

## Conflict of Interest

The authors declare that the research was conducted in the absence of any commercial or financial relationships that could be construed as a potential conflict of interest.

## Publisher's Note

All claims expressed in this article are solely those of the authors and do not necessarily represent those of their affiliated organizations, or those of the publisher, the editors and the reviewers. Any product that may be evaluated in this article, or claim that may be made by its manufacturer, is not guaranteed or endorsed by the publisher.
